# Analysis of immune responses induced by avian pathogenic *Escherichia coli* infection in turkeys and their association with resistance to homologous re-challenge

**DOI:** 10.1186/1297-9716-45-19

**Published:** 2014-02-14

**Authors:** Jean-Rémy Sadeyen, Pete Kaiser, Mark P Stevens, Francis Dziva

**Affiliations:** 1 The Pirbright Institute, Compton, Newbury, Berkshire RG20 7NN, UK; 2 The Roslin Institute and Royal (Dick) School of Veterinary Studies, University of Edinburgh, Easter Bush, Midlothian EH25 9RG, UK; 3 Present address: School of Veterinary Medicine, University of the West Indies, St Augustine, West Indies, Trinidad and Tobago

## Abstract

Avian pathogenic *Escherichia coli* (APEC) cause severe respiratory and systemic disease in poultry yet the nature and consequences of host immune responses to infection are poorly understood. Here, we describe a turkey sub-acute respiratory challenge model and cytokine, cell-mediated and humoral responses associated with protection against homologous re-challenge. Intra-airsac inoculation of turkeys with 10^5^ colony-forming units of APEC O78:H9 strain *χ*7122nal^R^ induced transient and mild clinical signs of colibacillosis followed by clearance of the bacteria from the lungs and visceral organs. Upon re-challenge with 10^7 ^*χ*7122nal^R^, primed birds were solidly protected against clinical signs and exhibited negligible bacterial loads in visceral organs, whereas age-matched control birds exhibited high lesion scores and bacterial loads in the organs. Levels of mRNA for signature cytokines suggested induction of a Th1 response in the lung, whereas a distinct anti-inflammatory cytokine profile was detected in the liver. Proliferative responses of splenocytes to either Concanavalin A or soluble *χ*7122nal^R^ antigens were negligible prior to clearance of bacteria, but APEC-specific responses were significantly elevated at later time intervals and at re-challenge relative to control birds. Primary infection also induced significantly elevated *χ*7122nal^R^-specific serum IgY and bile IgA responses which were bactericidal against *χ*7122nal^R^ and an isogenic Δ*rfb* mutant. Bactericidal activity was observed in the presence of immune, but not heat-inactivated immune serum, indicating that the antibodies can fix complement and are not directed solely at the lipopolysaccharide O-antigen. Such data inform the rational design of strategies to control a recalcitrant endemic disease of poultry.

## Introduction

Avian pathogenic *Escherichia coli* (APEC) are a leading cause of morbidity and mortality in poultry and exert significant economic and welfare costs. Infections are frequently associated with sudden death, salpingitis, peritonitis, pericarditis, perihepatitis, airsacculitis and with reduced yield, quality and hatching of eggs. Analysis of the repertoire of virulence-associated genes among APEC has indicated that some strains are closely related to *E. coli* causing human extraintestinal infections, in particular uropathogenic *E. coli* and neonatal meningitis-causing *E. coli*, indicating that they may also pose a threat of zoonosis [1,2]. Serogroups O1, O2 and O78 are commonly associated with avian colibacillosis; however, many other serogroups have been isolated from diseased birds and the prevalence varies with geographical location reviewed in [3,4]. Respiratory viral or *Mycoplasma* infections, the onset of sexual maturity and stress associated with sub-optimal husbandry practices are leading antecedents to colibacillosis in farmed poultry. Despite improvements in poultry production systems over the years, APEC continue to pose a formidable challenge to poultry farmers, threatening food security at a time of increasing global demand. The expansion of free-range production systems in Europe and elsewhere may be expected to increase the incidence of colibacillosis owing to greater exposure of farmed birds to environmental pathogens, stress and injury associated with formation of a social hierarchy. Indeed, in field surveys colibacillosis was the most common bacterial infection in birds reared free-range (with cases peaking between onset of lay and 30 weeks of age) and a positive correlation between vent-pecking and the incidence of colibacillosis was reported [5,6].

Systemic APEC infections are believed to arise from colonisation of the lower respiratory tract following inhalation of contaminated faecal dust, where levels can reach up to 10^6^ viable *E. coli* per gram in poultry houses [7]. Colonisation of the airsacs is enhanced by suppression of muco-ciliary activity and other upper respiratory tract defences resulting from concurrent infections and elevated ammonia levels in poultry houses. Avian airsacs are relatively avascular structures lacking effective resident defence mechanisms [8], hence control of pathogens is thought to be reliant upon recruitment of heterophils and macrophages [9]. The mode of translocation of APEC from the respiratory tract to the bloodstream is ill-defined. APEC can be found in the bloodstream as early as 3 hours after intra-airsac inoculation of naïve birds [10,11], and the immune responses that constrain such spread are unclear. Systemic spread of APEC may be followed by sepsis or localised inflammation in survivors involving extensive heterophil infiltration in organs of the reticulo-endothelial system [12-14].

Prophylactic use of antibiotics to control APEC in poultry is restricted owing to the risk of residues entering the food chain and the potential for the evolution of multi-drug resistant strains. Vaccination offers an attractive route to control APEC and inactivated and live-attenuated vaccines are commercially available. Autologous bacterins are effective but confer serogroup-specific protection and are believed to act principally through induction of humoral responses. The serogroup-specificity of such vaccines has been inferred to be due to the dominance of responses to the lipopolysaccharide O antigen. As avian colibacillosis is caused by multiple APEC serotypes, a requirement exists for broadly cross-protective vaccines. Earlier studies indicate that live-attenuated APEC vaccines or a low dose of virulent APEC confer a higher degree of cross-serogroup protection compared to killed vaccines [15,16], however, the immunological basis of protection has not been properly dissected. Although passively-administered hyperimmune serum conferred protection in intact birds [15,17,18], the contribution of innate defenses and cell-mediated immunity to the control of APEC in the avian host remains ill-defined. A better understanding of the nature and consequences of avian responses to APEC infection, and their association with recovery from primary infection and protection against re-challenge, can be expected to inform the rational design of control strategies.

In this study, we developed a model of sub-acute APEC O78:H9 infection in turkey poults and examined cytokine and antigen-specific cell-mediated and humoral responses prior to and after homologous re-challenge.

## Materials and methods

### Bacterial strains, media and growth conditions

APEC strain *χ*7122nal^R^ was obtained from Professor John Fairbrother (University of Montreal, Quebec, Canada) and belongs to serotype O78:H9. Strain *χ*7122 was originally isolated from the liver of a turkey with fatal colibacillosis [19]. Strain *χ*7122nal^R^ is a spontaneous nalidixic acid-resistant derivative of the original strain [20], which has been fully sequenced and used in studies on the molecular basis of virulence of APEC in turkeys [21]. Strain *χ*7145nal^R^ is an isogenic Δ*rfb* derivative of *χ*7122nal^R^ lacking the lipopolysaccharide O antigen [20]. The strains were stored in Luria-Bertani (LB) broth containing 15% (v/v) glycerol at -80 °C. For preparation of inocula, an overnight LB broth culture of strain *χ*7122nal^R^ was harvested by centrifugation, washed three times in sterile physiological saline and re-suspended to a volume equating to ca. 10^6^ colony-forming units (CFU) per mL for primary challenge or ca. 10^8^ CFU/mL for re-challenge. Bacteria were enumerated by retrospective plating of serial ten-fold dilutions of inocula or tissue homogenates using MacConkey agar (Oxoid, Basingstoke, UK) supplemented with 25 μg/mL of nalidixic acid (Mac + Nal). If required, prior enrichment was performed in brain heart infusion (BHI) broth overnight at 37 °C.

### Experimental animals

Animal experiments were conducted according to the requirements of the Animal (Scientific Procedures) Act 1986 (licence no. 30/2463) with the approval of the Local Ethical Review Committee. Ninety male Big5FLX turkeys were obtained from Aviagen Ltd. (Tattenhall, Cheshire, UK) as day-old poults. The poults were housed in biosecure accommodation to specific pathogen-free status on floor litter according to the guidelines issued by Aviagen Ltd. to simulate commercial practice. Water and a standard turkey feed ration were provided *ad libitum* for the duration of the experiment.

### Primary and secondary infection with APEC strain *χ*7122nal^R^

At two weeks of age, birds were divided into two groups (45 birds each) and housed separately. The first group was inoculated with 10^5^ CFU of washed stationary phase *χ*7122nal^R^ in 100 μL of saline by injection into the left caudal thoracic airsac and the second control group received 100 μL of sterile saline at the same site. Birds were closely monitored (at least every 3 h) for signs of disease for 3 days post-infection (dpi) and thereafter twice daily for the duration of the experiment. On days 1, 3, 5, 7, 14, 21 and 28 after primary infection, five birds were randomly removed from each group, killed humanely and subjected to post-mortem analysis. From each bird, pieces of liver and lung were collected in RNAlater (Ambion, Warrington, UK) for cytokine analysis, and the remainder was saved for enumerating bacteria. The spleen was collected into ice-cold RPMI1640 medium for T cell re-stimulation assays and blood and bile were collected for serology.

On day 41 post-primary challenge, all the remaining birds (10 in each group) were inoculated into the right caudal thoracic airsac with 10^7^ CFU of strain *χ*7122nal^R^ in 100 μL of saline and monitored overnight for clinical signs. After 24 h, all the birds were killed humanely and subjected to post-mortem analysis. The appearance of lesions defining acute colibacillosis (airsacculitis, perihepatitis, pericarditis and fibrin deposits on serosal surfaces) was recorded. Part of the spleen was collected for T cell proliferation assays. Lung, liver, kidney and the remaining spleen tissue were sampled to enumerate bacteria and blood and bile were collected for serology.

### Enumeration of viable APEC strain *χ*7122nal^R^ in tissues

Viable tissue-associated bacteria were enumerated essentially as described [21]. Briefly, 1 g of tissue was weighed and mixed with 9 mL of sterile phosphate-buffered saline (PBS) and suspended with an UltraTurrax homogenizer (IKA®Werke, Staufen, Germany) at room temperature to obtain a 1:10 dilution. Serial 10-fold dilutions were prepared in PBS and plated in triplicate on Mac + Nal agar and incubated at 37 °C overnight. The theoretical lower limit of detection by this method was 2 log_10_ CFU per gram. Enrichment cultures were also performed in which 1 mL of tissue homogenate was cultured in 9 mL BHI broth, to give a theoretical lower limit of detection of 1 log_10_ CFU per gram. Selected nalidixic acid resistant colonies were analysed by multiplex polymerase chain reaction (PCR) for virulence-associated genes of APEC (*cvaC*, *iss*, *iucD* and *tsh*) using oligonucleotide primers listed in Table 1 and by slide agglutination with O78-specific antiserum (Veterinary Laboratories Agency, Weybridge, UK) to confirm re-isolation of the challenge strain.

**Table 1:** Real-time quantitative RT-PCR probes and primers for turkey cytokines and primers for APEC genes used in this study

**Target**	**Primer/Probe**	**Sequence (5′- 3′)**	**Species**	**Reference**
**Cytokines**				
28S	Probe	(FAM)-AGGACCGCTACGGACCTCCACCA-(TAMRA)	C, T	[22]
	Forward	GGCGAAGCCAGAGGAAACT		
	Reverse	GACGACCGATTTGCACGTC		
IL-1β	Probe	(FAM)-CCACACTGCAGCTGGAGGAAGCC-(TAMRA)	C, T	[22]
	Forward	GCTCTACATGTCGTGTGTGATGAG		
	Reverse	TGTCGATGTCCCGCATGA		
CXCLi2	Probe	(FAM)-TCTTTACCAGCGTCCTACCTTGCGACA-TAMRA	C,T	[23]
	Forward	GCCCTCCTCCTGGTTTCAG		
	Reverse	TGGCACCGCAGCTCATT		
IFN-γ	Probe	(FAM)-AAAGATATCATGGACCTGGCCAAGCTTCA-(TAMRA)	T	[23]
	Forward	AACCTTCCTGATGGCGTGAA		
	Reverse	CTTGCGCTGGATTCTCAAGTC		
IL-13	Probe	(FAM)-TGCCAGCTGAGCACCGACAACG-(TAMRA)	T	[23]
	Forward	CCTGCACGGCCAGATGA		
	Reverse	GGCAAGAAGTTCCGCAGGTA		
IL-10	Probe	(FAM)-CCTGAAGATGACAATGAAGCGCTGTCA-(TAMRA)	T	[23]
	Forward	CGACCTGGGCAACATGCT		
	Reverse	CCTCTCGCAGGTGAAGAAGTG		
TGF-β4	Probe	(FAM)-ACCCAAAGGTTATATGGCCAACTTCTGCAT-(TAMRA)	C, T	[23]
	Forward	AGGATCTGCAGTGGAAGTGGAT		
	Reverse	CCCCGGGTTGTGTTGGT		
**APEC genes**				
*iss*	Forward	ATCACATAGGATTCTGCCG	-	[24]
	Reverse	CAGCGGAGTATAGATGCCA		
*iuc*D	Forward	ACAAAAAGTTCTATCGCTTCC	-	[25]
	Reverse	CCTGATCCAGATGATGCTC		
*cvi*/*cva*C	Forward	TGGTAGAATGTGCCAGAGCAAG	-	[24]
	Reverse	GAGCTGTTTGTAGCGAAGCC		
*tsh*	Forward	ACTATTCTCTGCAGGAAGTC	-	[24]
	Reverse	CTTCCGATGTTCTGAACGT		

C = chicken, T = turkey.

### Total RNA extraction and real-time quantitative RT-PCR for cytokine mRNA

Approximately 15 mg of lung and liver sampled at the time intervals indicated above were separately homogenized in 600 μL of RLT lysis buffer (Qiagen, Crawley, UK) using a bead mill (Retsch MM300, Retsch, Haan, Germany) and Qiashredders (Qiagen). Total RNA was extracted from homogenates using an RNeasy mini kit (Qiagen) following the manufacturer’s instructions. Purified RNA was eluted with RNase-free water and stored at -20 °C prior to use.

TaqMan real-time quantitative reverse transcriptase polymerase chain reaction (qRT-PCR) was used to quantify mRNA levels of a proinflammatory cytokine (IL-1β), a proinflammatory chemokine (CXCLi2), a Th1 signature cytokine (interferon (IFN)-γ), a Th2 signature cytokine (interleukin (IL)-13) and two T regulatory cytokines (IL-10 and TGF-β4), with 28S rRNA as a housekeeping gene, essentially as described [26,27]. Primers and probes specific for turkey mRNAs are given in Table 1. Briefly, qRT-PCR was performed using the Master Mix RT-PCR kit (Eurogentec, Seraing, Belgium). Amplification and detection of specific products were performed using the Applied Biosystems 7500 Fast Real-Time PCR System with the following cycle profile: one cycle at 48 °C for 30 min and 95 °C for 20 s, followed by 40 cycles at 95 °C for 3 s and 60 °C for 30 s. Quantification was based on increased fluorescence detected due to hydrolysis of the target-specific probes by the 5′exonuclease activity of the *rTth* DNA polymerase during PCR amplification. The passive reference dye 6-carboxy-c-rhodamine, not involved in amplification, was used for normalisation of the reporter signal. Data are expressed in terms of the cycle threshold value (Ct), normalised for each sample using the Ct value of 28S rRNA product for the same sample, as described previously [26,27]. Final results are shown as corrected ΔCt, using the normalised value.

### Preparation of soluble APEC antigens and O78 lipopolysaccharide

LB broth (100 mL) containing 25 μg/mL nalidixic acid was inoculated with APEC strain *χ*7122nal^R^ and incubated in a 250 mL Erlenmeyer flask overnight with shaking at 37 °C. Stationary phase bacteria were collected by centrifugation at 4000 × *g* for 30 min at 4 °C, washed 3 times, re-suspended in 2 × 10 mL PBS and stored on ice. Bacterial cells were disrupted by sonication (for a total of 2 min with 20 s cooling intervals using a Sonicator XL ultrasonic processor (Heat Systems Inc., Farmingdale, NY, USA). Cell debris was removed by centrifugation (1000 × *g* for 10 min) at 4 °C. The supernatant was further centrifuged at 30 000 × *g* for 30 min at 4 °C to remove the insoluble fraction. The supernatant containing soluble APEC antigens was collected and kept on ice. The total protein concentration was determined by a colorimetric assay using the bicinchoninic acid method (Pierce Biotechnology Inc., Rockford, IL, USA) before the soluble fraction was dispensed into single-use aliquots and stored at -70 °C until required.

O78 lipopolysaccharide (LPS) was purified from 2 mL of an overnight culture of strain *χ*7122nal^R^ (OD_600_ = 1.2) using a commercial LPS extraction kit (Chembio Ltd, Hertfordshire, UK) as per the manufacturer’s protocol. Approximately 30 μg of purified LPS was obtained from 2 mL culture and stored at 4 °C prior to use. Purity and integrity of LPS was confirmed by silver-staining of extracts resolved by sodium dodecyl sulphate polyacrylamide gel electrophoresis (SDS-PAGE).

### Isolation of splenocytes and T cell proliferation assays

Single splenocyte suspensions were prepared essentially as described [28]. Briefly, spleen tissue was passed through a Falcon cell strainer (BD Biosciences, Oxford, UK) in RPMI1640 medium (GibcoBRL, Paisley, UK) supplemented with 100 U/mL penicillin, 1 μg/mL streptomycin and 5% (v/v) foetal calf serum. The majority of red blood cells were removed by gentle centrifugation at 35 × *g* for 10 min. The supernatant primarily comprised lymphocytes, a few red blood cells and accessory cells as assessed by light microscopy of a Giemsa-stained smear. The cells were adjusted to a concentration of 5 × 10^6^ cells/mL in RPMI1640 supplemented with 5% (v/v) foetal calf serum and dispensed into micro-titre plates in 100 μL per well. For re-stimulation, 5 μg/mL of APEC soluble antigen or 4 μg/mL of Concanavalin A were added per well as required. When necessary, splenocytes were stimulated with 0.3 μg of purified LPS serogroup O55:B5 (Sigma, St Louis, MO, USA). After 24 h of incubation at 41 °C, 5% CO_2_, the cell suspensions were pulsed with 1 μCi^3^H-thymidine (Amersham, Little Chalfont, UK) per well. The plates were incubated for a further 16 h at 41 °C before harvesting with a Tomtec Mach IIIM cell harvester (Receptor Technologies, Banbury, UK). Incorporation of ^3^H-thymidine was measured using a 1450 Microbeta Trilux Scintillation counter (Perkin-Elmer, Beaconsfield, UK) and is expressed as mean counts per minute (cpm).

### Enzyme-linked immunosorbent assay (ELISA)

Nunc 96-well ELISA plates were coated with 1 μg of soluble APEC antigen or 0.6 μg of purified O78 LPS in 100 μL of coating buffer (KPL Inc., Gaithersburg, MD, USA) overnight at 4 °C. Excess antigen was removed by washing the Plates 3 times in PBS supplemented with 0.05% (v/v) Tween-20 (PBS-T). The plates were blocked with PBS-T containing 5% (w/v) skimmed milk (blocking buffer) for 1 h at room temperature with gentle agitation and then washed once in PBS-T. Pre-determined sample dilutions of serum (1:640) or bile (1:2) in PBS-T plus 0.5% (w/v) skimmed milk were added to antigen-coated wells and incubated at room temperature for 1 h with gentle agitation. Excess antibodies were removed by washing 3 times in PBS-T. Plates containing serum were incubated with a 1:2000 dilution of goat anti-turkey IgY (H + L) horse-radish peroxidise conjugate (KPL Inc., Gaithersburg, Maryland, USA) whilst plates containing bile were incubated with a 1:10 000 dilution of goat anti-chicken IgA (Serotec, Oxford, UK) at room temperature for 1 h. After removal of excess secondary antibodies, tetramethylbenzidine substrate (TMB, Sigma) was added and the plates incubated in the dark at room temperature for 10 min. Colour formation was stopped with 2 M sulphuric acid. The absorbance was read at 450 nm (OD_450_) with a background correction of 630 nm.

### In vitro bactericidal activity of immune and non-immune sera

To assess bactericidal activity of APEC-specific antibodies, *E. coli* strains *χ*7122nal^R^ and *χ*7145nal^R^ (an isogenic Δ*rfb* mutant) were cultured in sera derived from challenged and control birds obtained at day 42 as described [29]. Briefly, overnight broth cultures were sub-cultured in fresh LB broth, grown to mid-logarithmic phase, harvested, washed and adjusted in PBS to approximately 10^8^ CFU per mL. For each strain, 100 μL of bacterial suspension was separately mixed with 900 μL of immune serum, non-immune serum or LB broth (positive control) and incubated statically at 37 °C. Bacterial counts were determined by plating serial ten-fold dilutions of 20 μL sampled from each mixture at 0, 1, 2 and 3 h of incubation. When required, complement was inactivated by incubating sera at 56 °C for 1 h prior to the assays. The data presented for each strain are the mean of three independent assays performed in triplicate per serum sample.

### Statistical analyses

Statistical analysis of data from splenocyte re-stimulation and ELISA assays for infected and control groups was carried out using a Mann–Whitney U-test [30]. For the cytokine analyses, a one-way analysis of variance (ANOVA) was performed to detect differences between mean values, which were then analysed further for significance with Tukey’s test. Bacterial counts from tissues were analysed using a Student’s *t*-test. Bacterial survival in sera was analysed by an F-test, with the bacterial counts taken as repeated measurements and the growth medium as a covariant (Proc Mixed, Statistical Analysis System 1995, SAS Institute, Cary, NC, USA). In all cases, *P* values ≤ 0.05 were considered significant.

## Results

### Development of a turkey model of sub-acute primary APEC O78:H9 infection

**Figure 1:**
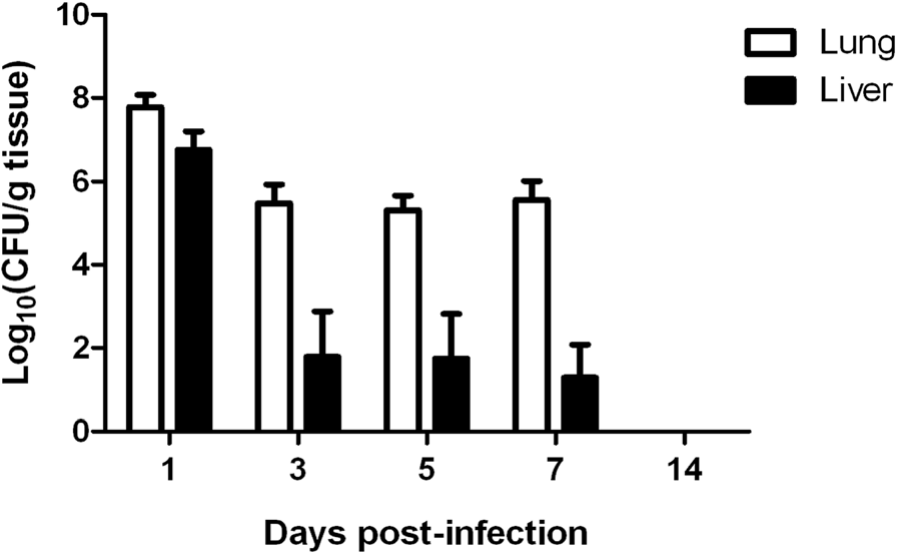
**Recoveries of nalidixic acid-resistant *E. coli *after primary infection.** Two-week-old turkey poults were given 10^5^ CFU of APEC O78:H9 strain *χ*7122nal^R^ via the intra-airsac route. Five birds were randomly selected, humanely killed and lung (open bars) and liver (shaded bars) sampled for enumeration of bacteria at each time-point. The data represent the mean log_10_CFU/g tissue ± standard error (SE) of the mean.

Intra-airsac inoculation of two-week-old turkey poults with 10^5^ CFU of APEC O78:H9 strain *χ*7122nal^R^ resulted in mild clinical signs (ruffled feathers, hunched posture and depression) which resolved within 24 h in all the challenged birds. Such clinical signs were absent in the control group that received sterile saline via the same route. After 24 h, APEC-challenged birds remained normal in appearance for the duration of the study. Five birds were randomly sampled per group to determine the level of bacterial colonisation, the degree of pathology and the induction of splenocyte responses. At post-mortem examination, pathological lesions including all or one of; airsacculitis, pericarditis, perihepatitis and fibrin deposits on serosal surfaces were observed in all APEC-infected birds killed at days 1 and 3 post-infection (pi) (Table 2), but not in control birds. Enumeration of tissue-associated APEC revealed ca. 10^7^ CFU nalidixic acid-resistant *E. coli* per gram of lung and liver tissues 1 day after infection (Figure 1). The number of birds exhibiting lesions decreased with time (Table 2) consistent with the declining numbers of nalidixic acid- resistant *E. coli* recovered from the tissues (Figure 1). All colonies examined were shown to possess the O78 antigen and genes found in *χ*7122nal^R^ but not most avian faecal *E. coli*. No *E. coli* were isolated from the organs of any bird in the control group at any time. At day 14 pi, none of the APEC-infected birds yielded bacteria from either the lung or liver by direct plating or enrichment, implying that they had cleared the infection. Minimal variance was detected in bacterial numbers in the organs sampled or clinical signs, reducing the likelihood of highly variable host responses.

**Table 2:** **The relationship between clinical signs, post-mortem lesions, recoveries of APEC strain** ***χ*** **7122nal**^**R**^**from lung and liver and splenocyte proliferation in response to** ***χ*** **7122nal**^**R**^**soluble antigen in the first 14 dpi**

**Parameter**	**Dpi**																								
	**Day 1**					**Day 3**					**Day 5**					**Day 7**					**Day 14**				
	**Bird number**					**Bird number**					**Bird number**					**Bird number**					**Bird number**				
	**1**	**2**	**3**	**4**	**5**	**6**	**7**	**8**	**9**	**10**	**11**	**12**	**13**	**14**	**15**	**16**	**17**	**18**	**19**	**20**	**21**	**22**	**23**	**24**	**25**
Clinical signs^1^	**+**	**+**	**+**	**+**	**+**	**-**	**-**	**-**	**-**	**-**	**-**	**-**	**-**	**-**	**-**	**-**	**-**	**-**	**-**	**-**	**-**	**-**	**-**	**-**	**-**
Post-mortem lesions^2^	**+**	**+**	**+**	**+**	**+**	**+**	**+**	**+**	**+**	**+**	**+**	**+**	**+**	**-**	**-**	**+**	**-**	**-**	**-**	**-**	**-**	**-**	**-**	**-**	**-**
Log_10_CFU APEC																									
Lung	8.9	7.6	7.2	7.5	7.6	6.1	5.2	4.2	5.1	6.8	5.3	4.2	6.4	5.4	5.3	5.2	4.2	7.0	5.8	5.6	0	0	0	0	0
Liver	8.3	7.1	6.5	5.9	6.1	4.7	4.2	1.0*	1.0*	1.0*	4.2	1.0*	4.5	1.0*	0	3.2	3.3	1.0*	0	0	0	0	0	0	0
Splenocyte proliferation^3^	**-**	**-**	**-**	**-**	**-**	**-**	**-**	**-**	**-**	**-**	**-**	**-**	**-**	**-**	**+**^**a**^	**-**	**-**	**-**	**+**^**a**^	**+**^**a**^	**+**^**b**^	**+**^**b**^	**+**^**b**^	**+**^**b**^	**+**^**b**^

^1^Depression, ruffled feathers, hunched posture.

^2^Airsacculitis, pericarditis, perihepatitis, fibrin on serosal surfaces; + = presence any of the clinical or pathological signs; - = absence of any observable clinical or pathological signs. None of these clinical signs or post-mortem lesions was seen in the control group of birds given sterile saline.

^3^Splenocyte proliferation, - = no response; +^a^ = counts between 200–250 cpm; +^b^ = counts ≥ 2000 cpm.

Bacterial recoveries from lung and liver are given as log_10_CFU. * = denotes a theoretical value of bacteria obtained after enrichment.

### Analysis of cytokine mRNA levels following primary APEC O78:H9 infection

**Figure 2:**
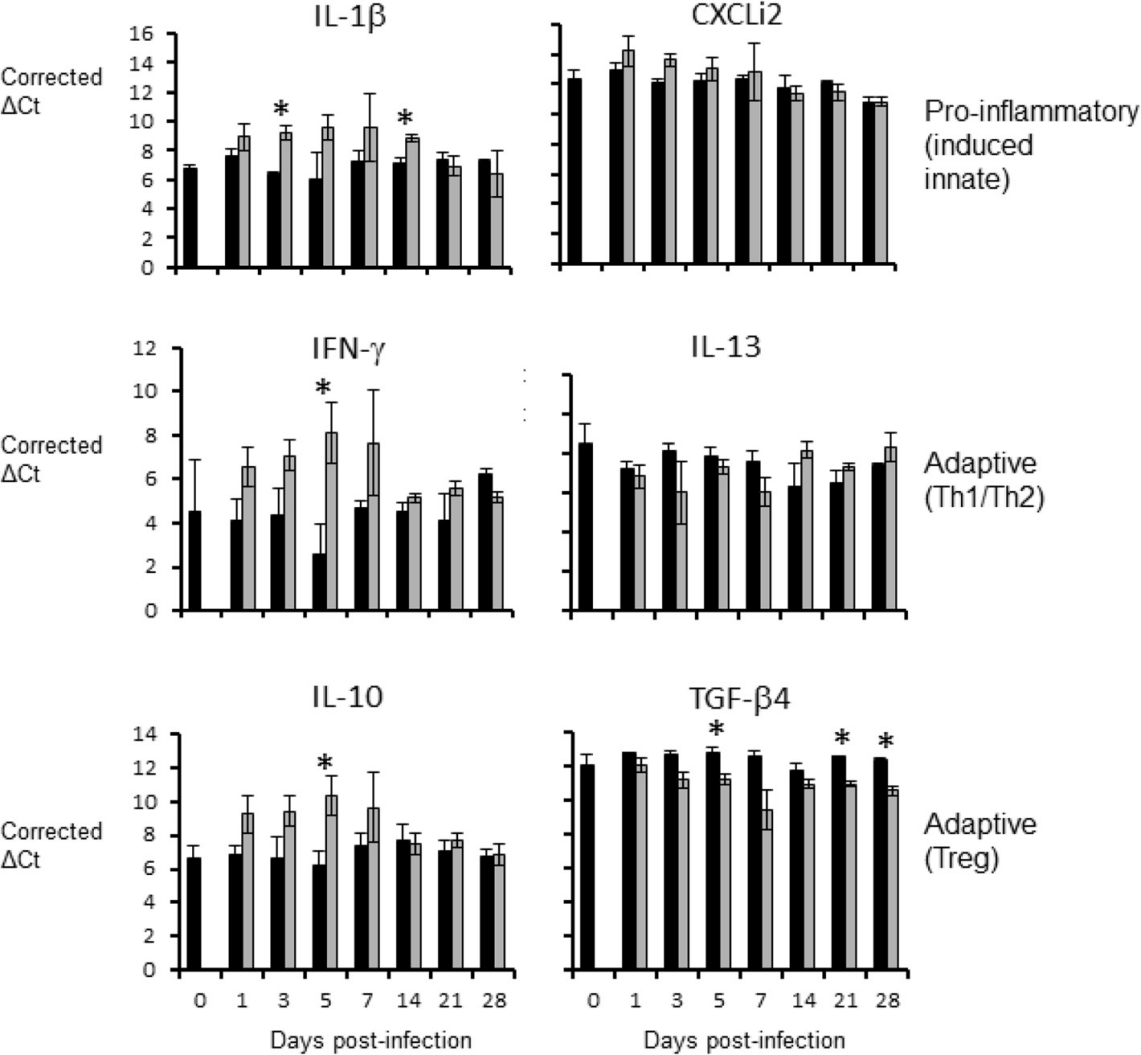
**Levels of cytokine mRNA transcripts in the lung as measured by real-time qRT-PCR (Taqman).** Results are expressed as corrected ΔCt values ± SE, in the lungs of challenged (grey bars) and age-matched control (black bars) chickens. Five challenged and three control birds were sampled per time-point. * = *P* ≤ 0.05.

**Figure 3:**
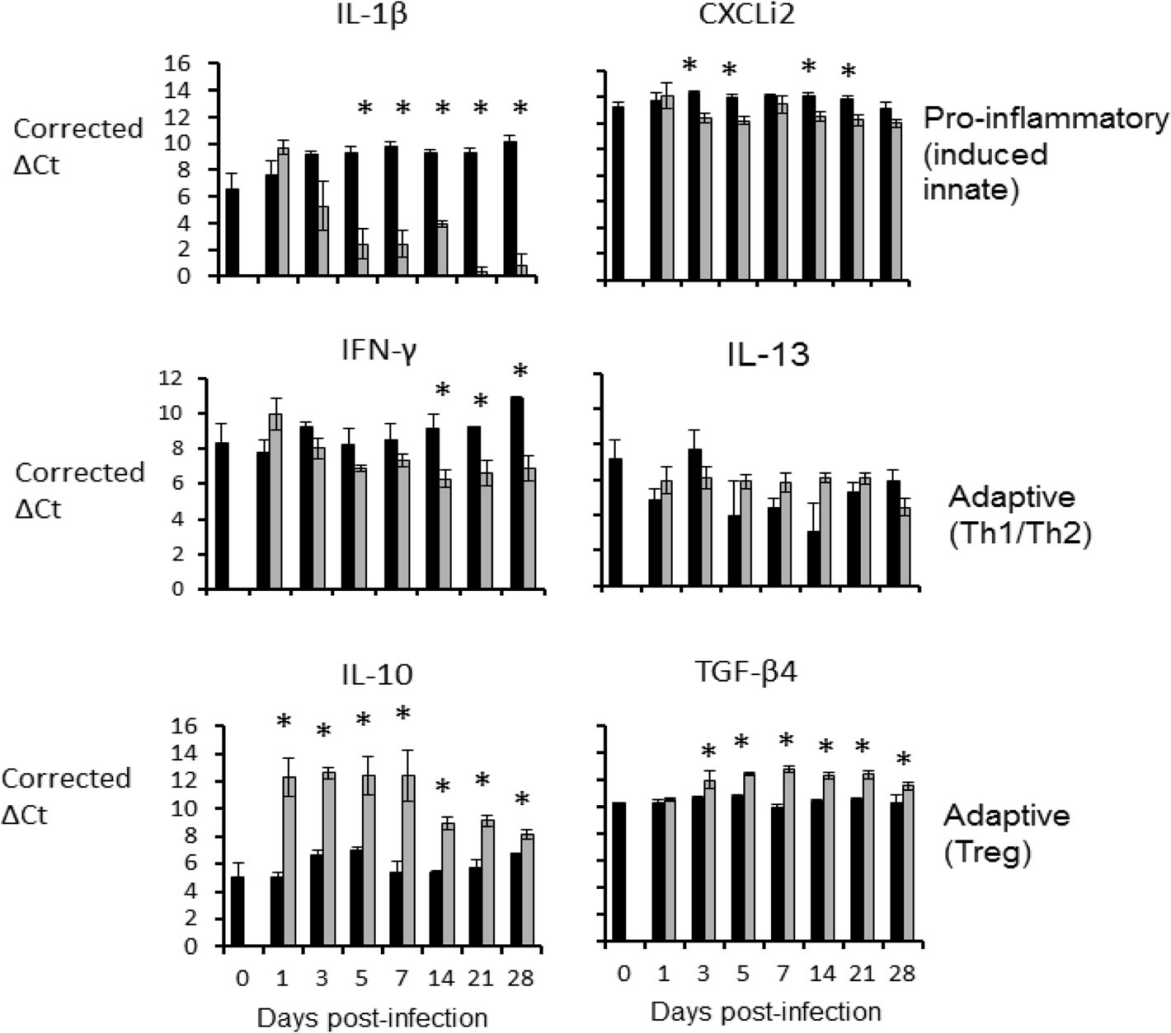
**Levels of cytokine mRNA transcripts in the liver as measured by real-time qRT-PCR (Taqman).** Results expressed as corrected ΔCt values ± SE, in the livers of challenged (grey bars) and age-matched control (black bars) chickens. Five challenged and three control birds were sampled per time-point.* = *P* ≤ 0.05.

Very different patterns of cytokine mRNA expression were seen in the lung and liver of APEC-infected birds. In the lung (Figure 2), cytokine mRNA levels were suggestive of a Th1 response. IL-1β mRNA levels were up-regulated in challenged birds, compared to levels in age-matched control birds, from days 1–14 pi, although only statistically significantly (*P* < 0.05) so at days 3 and 14. Levels of mRNA for IFN-γ, the signature Th1 cytokine, were up-regulated from days1-21 pi, but were statistically significantly different only at day 5. A mixed pattern was seen with the signature anti-inflammatory cytokines of a T regulatory response, IL-10 and TGF-β4. IL-10 mRNA expression levels were up-regulated in challenged birds from days 1–7 pi, statistically significantly so at day 5, whereas mRNA expression levels of TGF-β4 were down-regulated post-challenge throughout the experiment, with statistically significant differences at days 5, 21 and 28. In the liver (Figure 3), cytokine mRNA expression levels were suggestive of an anti-inflammatory response. In general, mRNA expression levels of the pro-inflammatory innate-induced cytokines IL-1β and CXCLi2 were down-regulated following challenge (statistically significant from day 5–28 pi for IL-1β, and days 3, 5, 14 and 21 pi for CXCLi2). IFN-γ mRNA expression levels were also down-regulated from days 3–28 pi (statistically significant at days 14–28 pi). In contrast, IL-13 mRNA expression levels were up-regulated from day 5–28 pi, although none of the observed differences were statistically significant. The most obvious differences between challenged and control birds in the liver were seen for the T regulatory cytokines. Both were up-regulated post-challenge, with these differences being statistically significant for IL-10 throughout the experiment and for TGF-β4 from day 3 pi for the remainder of the experiment.

### Primed birds are solidly protected against homologous re-challenge

**Figure 4:**
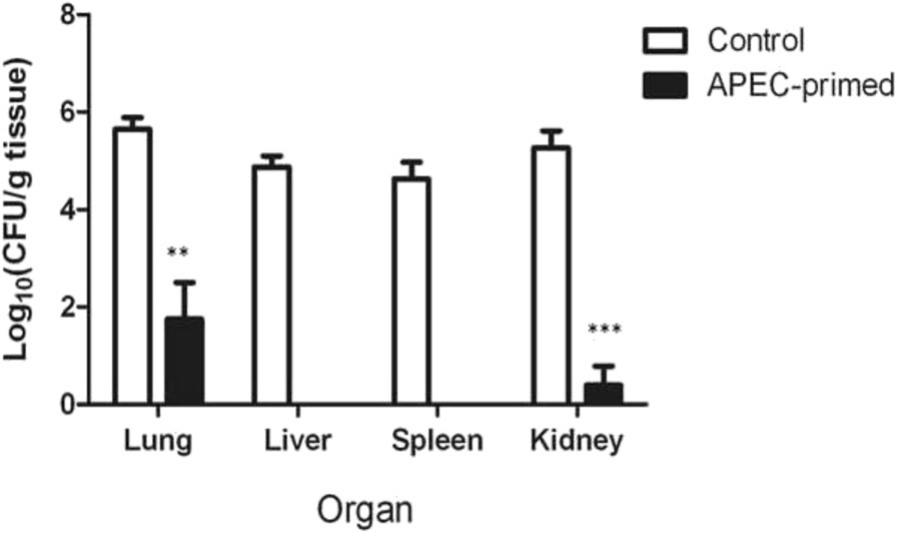
**Recoveries of nalidixic acid-resistant *E. coli *from internal organs after homologous re-challenge.** APEC-primed turkeys (*n* = 10) and their age-matched controls (*n* = 10) were challenged with 10^7^ CFU of APEC strain *χ*7122nal^R^ via the intra-airsac route and sampled after 24 h. The data represent the mean log_10_CFU/g tissue ± standard error of the mean.* = *P* ≤ 0.05; ** = *P* ≤ 0.01; *** = *P* ≤ 0.001.

To evaluate if primary infection could protect turkey poults against homologous re-infection, we inoculated 10 birds from the control and APEC-primed groups with 10^7^ CFU of strain *χ*7122nal^R^ on day 41 post-primary inoculation. At 24 h post-inoculation, 4/10 birds previously primed with APEC had ruffled feathers and brief periods of anorexia (which lasted no more than 4 h) and the remainder had no clinical signs, whilst the group that was previously mock-infected exhibited clinical signs of acute colibacillosis as defined above, as well as lesions typical of acute colibacillosis at post-mortem examination (Table 2). Consistent with such signs and lesions, significantly higher numbers of bacteria were recovered from visceral organs of birds in the control group compared to the APEC-primed group (Figure 4).

### Proliferative responses of splenocytes to APEC antigens after primary or mock infection

**Figure 5:**
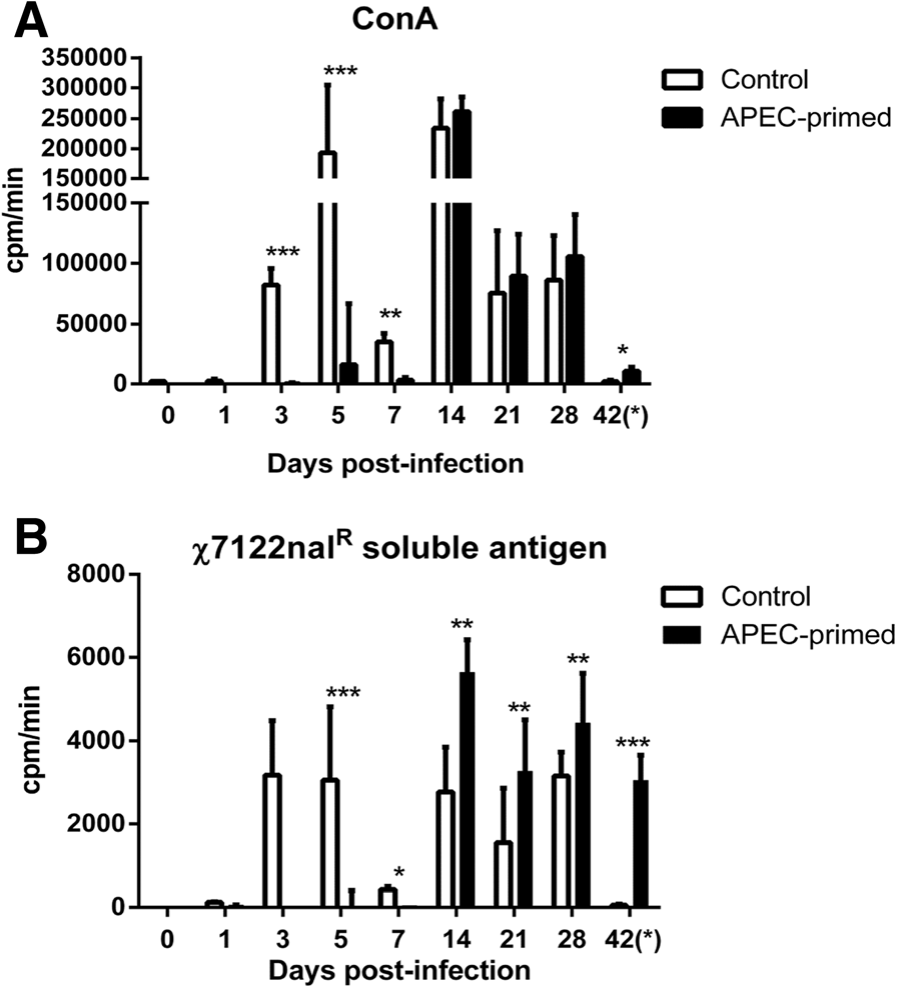
**Splenocyte proliferation of turkeys at time intervals after primary intra-airsac inoculation.** Turkey poults were given 10^5^ CFU of APEC strain *χ*7122nal^R^ and in uninfected controls were given sterile saline via the same route. Re-stimulation assays were also performed after challenge at day 42. Splenocyte suspensions were separately stimulated with Concanavalin A (positive control; **A)** and strain *χ*7122nal^R^ soluble antigen **(B)**. Five birds were sampled from each group at each interval.* = *P* ≤ 0.05; ** = *P* ≤ 0.01; *** = *P* ≤ 0.001.

To evaluate whether primary infection induced cellular immune responses specific to APEC antigens, we performed ex vivo stimulation assays on splenocytes from infected and control birds at the same intervals when bacterial counts were quantified in the lung and liver. In APEC-infected birds, the mean proliferation of splenocytes in response to Concanavalin A or soluble antigens from *χ*7122nal^R^ was low or absent on days 1, 3, 5 and 7 pi compared to their mock-infected counterparts, albeit that variation in the response of splenocytes from control birds existed at these times (Figures 5A-B), possibly arising from the out-bred nature of the birds. Statistically significant proliferative responses to *χ*7122nal^R^ soluble antigen were observed in infected birds relative to the control group from day 14 pi onwards, coinciding with a time-point when *χ*7122nal^R^ was not recovered from tissues. Such responses were absent in the control group after re-challenge (day 42 pi) consistent with the recovery of high numbers of *χ*7122nal^R^ in tissues including the spleen.

### Primary APEC infection elicits antigen-specific IgY and IgA responses

**Figure 6:**
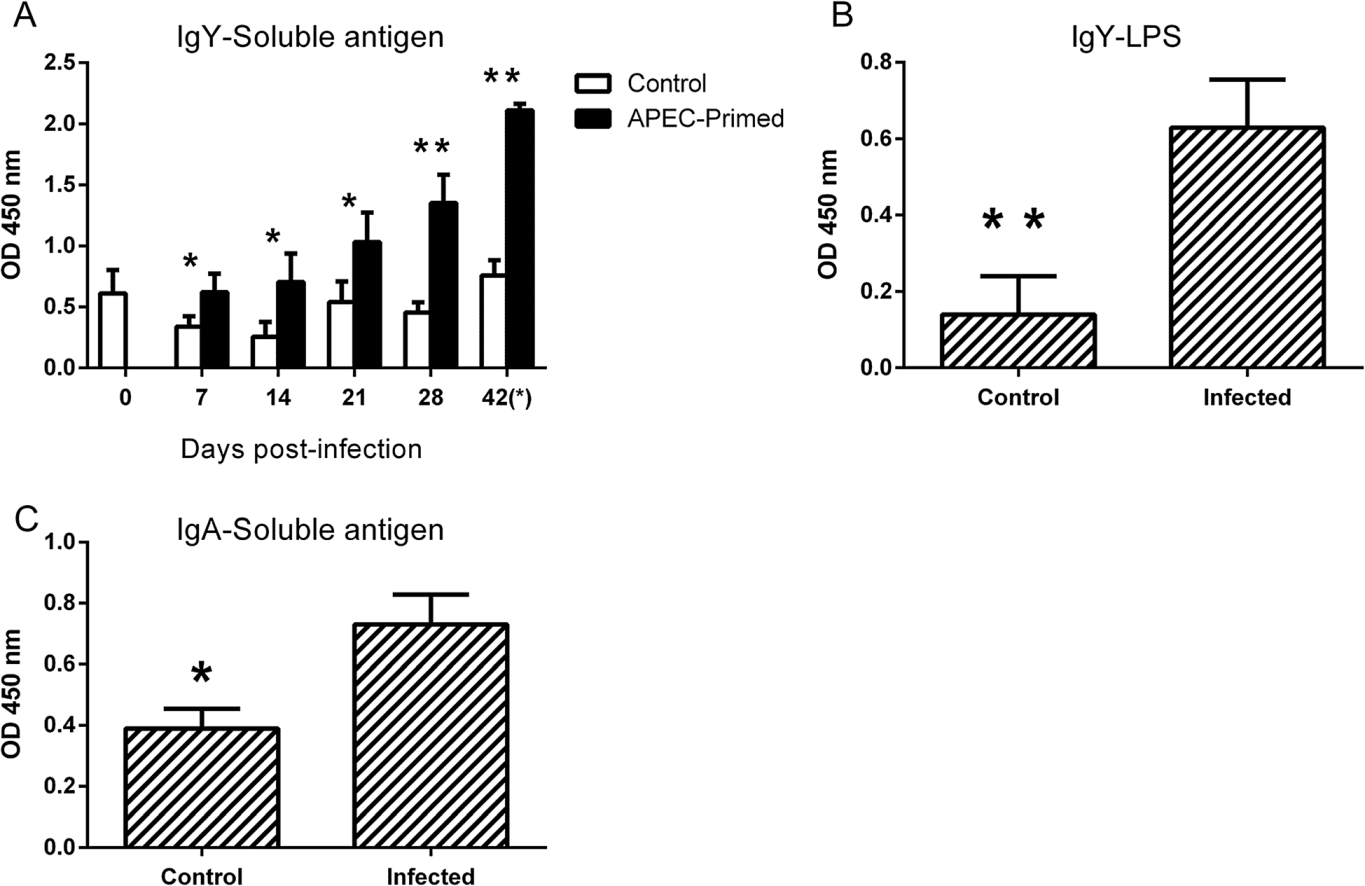
**Antibody levels in APEC- and mock-infected birds following primary infection and after homologous re-challenge.** The levels were measured by ELISA using different antigens; IgY levels against soluble *χ*7122nal^R^ antigens **(A)**, IgY levels against O78 LPS purified from *χ*7122nal^R^**(B)** and IgA levels measured against soluble *χ*7122nal^R^ antigens **(C)**. At 0, 7, 14, 21, and 28 days post-primary infection, five birds per group were sampled and ten birds per group were sampled after re-challenge (day 42). The data in panels B and C reflect levels at post-mortem examination on day 42, one day after re-challenge. * = *P* ≤ 0.05; ** = *P* ≤ 0.01.

To determine if resistance to homologous re-challenge could be associated with humoral responses, we measured APEC-specific serum IgY and bile IgA levels. Protected birds had significantly higher levels of serum IgY antibodies specific to both the *χ*7122nal^R^ soluble antigen and O78 LPS compared to the control group (Figures 6A and B). Levels of APEC-specific IgY rose over time and were significantly different from mock-infected birds from 7 days post-primary infection (Figure 6A). A significantly higher level of IgA specific to *χ*7122nal^R^ soluble antigen was observed in bile samples from protected APEC-primed birds compared to control birds at post-mortem examination on day 42 (Figure 6C).

### Serum from protected birds is bactericidal in vitro

**Figure 7:**
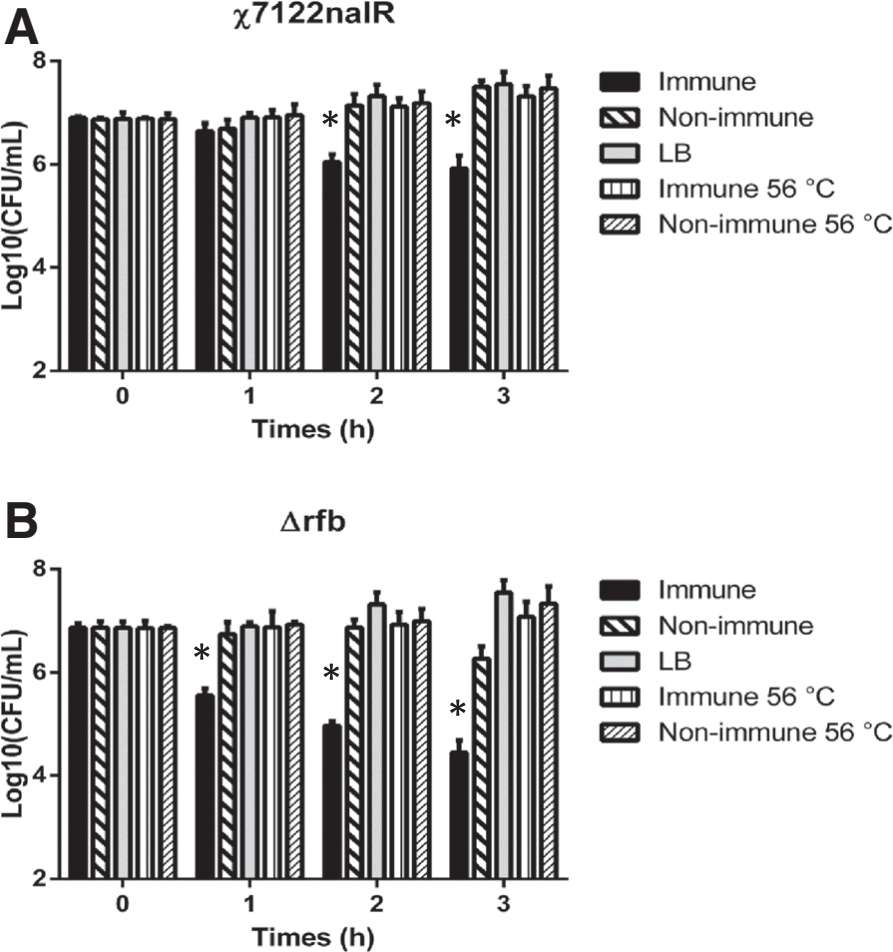
**Net replication of APEC strains in LB broth, immune and non-immune turkey sera.** Viable counts of *χ*7122nal^R^**(A)** and its isogenic Δ*rfb* mutant **(B)** were obtained over a 3 h incubation in immune, heat-inactivated immune, non-immune, heat-inactivated non-immune and LB broth. Bacterial counts were determined by sampling 20 μL of culture and performing serial 10-fold dilutions before plating on Mac + Nal agar. The data represent the mean log_10_ CFU ± standard error from three independent assays performed in triplicate.* = *P* ≤ 0.05.

To determine if immune serum was bactericidal, we performed in vitro assays to quantify antibody-directed complement-mediated killing. A log_10_-fold reduction in the number of viable bacteria was obtained 2–3 h after incubation with serum from birds infected with *χ*7122nal^R^ compared to that from control mock-infected birds (Figure 7A). To determine if this was a consequence of complement fixation, parallel assays were undertaken with heat-inactivated sera. Net replication of strain *χ*7122nal^R^ was similar in heat-inactivated immune and in non-immune sera (Figure 7A). On the other hand, the net growth of the isogenic Δ*rfb* mutant was mildly impaired by non-immune sera, but severely inhibited by immune serum (Figure 7B).

## Discussion

To study the immunological basis of clearance of primary APEC infection we elected to infect two-week-old turkey poults by the intra-airsac route, as previous studies using turkeys older than three weeks of age failed to elicit classical signs of colibacillosis and recoveries of APEC from tissues were low [13,31]. Mitogenic responses of turkey peripheral T cells develop rapidly after hatch and reach adult levels by two weeks-of-age [32], therefore we do not consider such birds to be immunologically immature. Whilst APEC may persist in the alimentary tract of healthy birds [33], the respiratory tract is considered a significant route of infection leading to systemic disease. Aerosol, intra-tracheal, intranasal and intra-airsac incoulation have been used to study APEC virulence factors, antibody-mediated passive protection, genetic susceptibility, efficacy of antimicrobial compounds and to evaluate candidate vaccines [10,11,15,34-36]. The merits and shortcomings of each of these respiratory infection models have been reviewed [37]. Inoculation of APEC into the airsac of healthy birds not only ensures that a standard dose is delivered at the site of natural infection [21], but circumvents upper respiratory mucosal defences that may reduce the number of bacteria that eventually reach this site. Strain *χ*7122nal^R^ was capable of producing severe clinical signs typical of acute colibacillosis, including ataxia, dyspnoea, rales and reduced response to stimulus, within 24 h of infection of naïve birds via the intra-airsac route with ca. 10^7^ CFU, and is therefore fully virulent [this study and 21]. A close association between the reduction in clinical/pathological signs and the declining numbers of recovered APEC obtained in this study (Table 2) is consistent with previous data in intra-airsac infection models [10,11]. The model described here therefore provides an opportunity to study the nature and consequences of adaptive immune responses following primary infection.

Cytokine responses elicited by APEC infection in the lung were different from those in the liver. Though inter-animal variation in responses of outbred birds limited conclusions to some extent, transcript levels for the signature Th1 cytokine IFN-γ were elevated at the site of primary infection on all days post-inoculation except day 28 pi, albeit only significantly so on day 5 pi. Induction of a Th1 response, though not formally proven by these data, would be consistent with the response expected for an intracellular pathogen. Though not statistically significant at all time-points sampled, transcripts encoding the pro-inflammatory cytokines IL-1β and CXCLi2 were up-regulated in the lung during the early phase of infection. In contrast, an anti-inflammatory response was seen in the liver. Such strong responses are unusual in poultry, let alone in the liver, especially when the bacteria were present in large numbers and clinical signs of infection were present. Consistent with this strong anti-inflammatory response, the regulatory cytokines IL-10 and TGF-β4 were up-regulated, being statistically significant at most time-points post-infection. It remains to be determined if these responses are driven by the host or by the pathogen.

Adaptive responses were measured to examine their association with protection against re-challenge. Though it is evident that APEC was rapidly controlled at the site of inoculation, it is unclear if the paucity of APEC in the liver, spleen and kidney of primed birds reflects control of bacteria that reached these organs, or absence of systemic translocation of the bacteria from the lung. Control of APEC at the site of inoculation could be partly attributed to elevated APEC-specific IgA levels, as detected in the bile of protected birds. We determined IgA levels in bile rather than the lung, owing to the possible interference of APEC at the site of infection with ELISA tests using lung washes, in accordance with published literature [38,39]. Rising titres of IgY were also seen in serum from day 7 onwards. It is possible that these humoral responses induced by primary infection are associated with resistance to re-challenge, as passive administration of hyperimmune serum from birds immunised with inactivated APEC O78 aided bacterial clearance from the blood and opsono-phagocytosis in the liver [15,17,18]. Furthermore, passively transferred egg-yolk antibodies conferred resistance against APEC respiratory tract infection [40]. To assess if such immune serum could be bactericidal in vitro, we compared the net replication of APEC in immune and non-immune sera. Consistent with published literature [41-44], strain *χ*7122nal^R^ was highly resistant to non-immune serum, with the net replication over time resembling that when grown in LB broth (Figure 7A), but decreasing in the presence of immune serum. A similarity in the net replication of bacteria in both heat-inactivated immune and non-immune sera suggested that APEC-specific antibody was predominantly enhancing complement-mediated lysis. The ability of immune serum to impair the net growth of an isogenic Δ*rfb* mutant indicates that antibody-mediated killing in the presence of complement is not strictly dependent on the O-antigen.

Cell-mediated immunity was assessed by measuring splenocyte responses to re-stimulation with strain *χ*7122nal^R^ soluble antigens. These responses were suppressed at sampling points when APEC was recovered from the liver and lung tissues (Figure 1). It is unclear whether the inhibition of mitogen-activated lymphocyte proliferation was a consequence of bacterial interference. To exclude the possibility that the lack of proliferative response was due to loss of cell viability, we performed a trypan blue dye exclusion test and confirmed splenocyte viability after stimulation (data not shown). Proliferative responses to the APEC antigens were seen in mock-infected birds at three days pi and subsequent time-points, suggesting non-specific stimulation or previous exposure to similar antigens. To test if this non-specific stimulation could have been caused by LPS (a non-specific mitogen), we separately stimulated splenocytes with purified LPS from *E. coli* serogoup O55:B5 and obtained a similar pattern of responses (data not shown). This suggested that the mock-infected birds could have been exposed to LPS of environmental or commensal *E. coli*. As earlier suggested, the lack of T cell responses in APEC-infected birds early after infection could be a consequence of bacterial interference. From day 14 onwards proliferative responses of splenocytes from the infected and control groups to soluble *χ*7122nal^R^ antigens were significantly different from each other (Figure 5B), suggesting the development of T cells specific to *χ*7122nal^R^ antigens. Such a pattern was not consistently evident in the ConA-stimulated splenocytes at these intervals (Figure 5A) since ConA is a non-specific mitogen. To the best of our knowledge, we are the first to report the induction of APEC-specific T cell responses after primary infection of turkeys and the significant elevation of such responses relative to control birds may be associated, though not formally correlated, with the resistance of primed birds to re-infection. The extent to which such APEC-specific T cells comprise effector or memory populations requires further study.

In conclusion, we describe a model of sub-acute APEC infection in turkeys in which a strain of a prevalent serogroup was rapidly cleared and poults solidly protected against homologous re-challenge, in a manner associated with the induction of both cell-mediated and humoral immunity. We recognise that further studies are required to establish the role of the responses observed, for example by passive transfer of antibody elicited by primary infection, bursectomy or adoptive transfer or depletion of cell populations. Our study, nevertheless, provides a valuable model to examine the nature and consequences of avian responses to a key endemic pathogen, and provides evidence of adaptive responses that could be exploited in the design of strategies for control of colibacillosis in poultry.

## Competing interests

The authors declare that they have no competing interests.

## Authors’ contributions

JRS and FD designed, conducted and supervised the experiments. FD, PK and MPS analysed and interpreted the data and wrote the manuscript. All authors read and approved the manuscript.
